# Cluster analysis of motor coordination and health-related fitness in preschool children: population classification and variable contribution based on principal component analysis

**DOI:** 10.3389/fpubh.2025.1630387

**Published:** 2025-09-24

**Authors:** Xiaoxiao Chen, Deqiang Zhao, Aoyu Zhang, Chunmiao Wang, Jin He, Jiaxin Chen, Haixia Hu, Xiaoni Tang, Aiying Zhang, Han Xiao, Yanfeng Zhang

**Affiliations:** ^1^China Institute of Sport Science, Beijing, China; ^2^Aiyumo Children and Youth Sports Health Research, Shandong, China; ^3^Changyi Experimental Kindergarten, Shandong, China; ^4^Weifang National Fitness Service Center, Shandong, China

**Keywords:** preschool children, motor coordination, health-related fitness, fitness index, correlation analysis, hierarchical regression

## Abstract

**Background:**

The preschool period is critical for children’s motor development and neural maturation. Fundamental motor skill development during this stage directly influences the nervous system’s growth. Motor coordination ability serves as the foundation for children’s physical fitness, health, and motor performance.

**Objective:**

This study investigates the relationship between motor coordination and health-related fitness in preschool children, analyzing the extent of motor coordination’s impact on physical fitness. Through principal component analysis (PCA) and cluster analysis, differences in motor coordination and fitness performance across child populations are identified, providing theoretical support for optimizing health promotion strategies.

**Methods:**

Participants included 358 preschool children from a kindergarten in Weifang, Shandong, China., including demographic data such as parental education, socioeconomic background, and habitual physical activity participation collected via parent questionnaires to provide contextual information Motor coordination was assessed using the Movement Assessment Battery for Children-2 (MABC-2), while fitness tests (e.g., standing long jump, tennis throw, 10-meter shuttle run) were standardized into Z-scores. Statistical analyses included Pearson correlation, hierarchical regression, PCA, and cluster analysis. PCA component retention was based on eigenvalues >1 and scree plot inspection, while cluster validity was confirmed using the elbow method and silhouette coefficients.

**Results:**

Motor coordination showed significant positive correlations with flexibility, strength, agility, and endurance. Hierarchical regression confirmed motor coordination’s independent and significant influence on fitness outcomes, particularly in standing long jump and shuttle run. BMI exhibited a weak negative correlation with motor coordination. PCA revealed two principal components (Dim1: 31.7% variance, dominated by running and jumping; Dim2: 16.1% variance, emphasizing flexibility and tennis throw). Cluster analysis categorized children into three groups: “Comprehensive Excellence,” “Agility Specialization,” and “Basic Skill Needs.”

**Conclusion:**

Motor coordination plays a pivotal role in preschool children’s fitness development, particularly in agility and strength. PCA and cluster analysis highlighted distinct group differences, supported by validated retention and validation procedures, underscoring the need for targeted interventions to enhance motor coordination and overall health.

## 1 Introduction

The preschool stage is a critical period for children’s physical, cognitive, fundamental motor, and social development. Among these domains, the acquisition of fundamental motor skills not only precedes advanced cognitive abilities such as numeracy and language but also peovides the foundation for their subsequent maturation (1). Motor skill development is reciprocally associated with the maturation of key brain regions, including the cerebellum, basal ganglia, and prefrontal cortex, which also underlie higher-order cognitive processes such as language, reasoning, and problem-solving. Evidence from animal models indicates that motor skill learning induces anatomical and physiological plasticity in the primary motor cortex (e.g., cortical map reorganization and synaptic changes) (2, 3), while neuroimaging and transcranial magnetic stimulation studies in humans demonstrate that motor training can expand cortical representations and enhance excitability of task-related muscle groups (4, 5). These findings highlight a bidirectional relationship: motor experiences promote neural development, while the state of neural maturation constrains motor learning (6). Consistent with this perspective, research has shown that motor skill acquisition depends on the maturation of the nervous system—for instance, a child cannot walk independently until neural structures are sufficiently developed—and that motor behavior reflects both physiological motor control and cognitive–behavioral learning processes working in concert (7). Consequently, motor coordination, defined as the ability to control and integrate body parts during simple and complex movements, represents an observable outcome of motor-cognitive integration, is closely tied to the maturation of the nervous, muscular, and musculoskeletal systems in preschool children, serving as a vital indicator of both physical skill progression and cognitive development (8) In this study, motor coordination was operationalized using the Movement Assessment Battery for Children-2 (MABC-2), a standardized tool with well-documented psychometric properties in preschool populations. Importantly, validation work in mainland China has demonstrated that the MABC-2 Age Band 1 possesses excellent inter-rater and test–retest reliability (ICC ≈ 0.90), acceptable internal consistency, and factorial validity, supporting its appropriateness for assessing motor coordination in Chinese preschool children (9).

With improvements in motor coordination, children’s physical fitness components (e.g., strength, endurance, flexibility, balance) develop synchronously. These components not only reflect advancements in motor abilities but also indirectly indicate overall health status (10). Studies have shown that children with stronger motor coordination typically exhibit higher motor proficiency and better health outcomes in physical activities. Their movements are more precise, fluid, and stable, enabling effective participation in various sports and fostering comprehensive physical development (11). Conversely, children with poor motor coordination may face greater health challenges. Uncoordinated movements can lead to physical fatigue, injuries, or disinterest in exercise. Repeated negative experiences may even foster resistance to physical activity, creating a detrimental cycle that impacts overall health (12).

Health-related fitness, a critical indicator of children’s health and physical competence, encompasses strength, endurance, flexibility, coordination, and other dimensions, directly reflecting preschool children’s motor performance and physical skill development (13). Good fitness not only helps prevent obesity and motor developmental delays but also positively influences mental health and holistic development (14). Regular exercise and structured physical training can effectively regulate body weight, improve body composition, reduce obesity risks, and enhance growth and learning efficiency (15). Research indicates that scientifically designed training programs such as aerobic activities (e.g., swimming, jogging, cycling) to improve cardiorespiratory endurance, and continuous or interval training to boost strength and endurance significantly enhance children’s fitness indices (16). Thus, the relationship between motor coordination and health-related fitness is closely intertwined, exerting a profound impact on children’s holistic health during the preschool period (17).

This study aims to explore the relationship between motor coordination and health-related fitness in preschool children, analyzing the influence of motor coordination on physical fitness development. Through empirical analysis, Pearson correlation and hierarchical regression will confirm significant associations between motor coordination and fitness indicators. Principal component analysis (PCA) will extract key components of fitness and motor coordination, while cluster analysis will identify distinct groups of children based on fitness performance. This framework facilitates a comprehensive understanding of the interplay between motor coordination and physical fitness, providing theoretical and practical insights for optimizing health promotion strategies and enhancing motor competence in preschool children.

During the preschool years, children undergo rapid development in physical, cognitive, and social skills, with motor coordination playing a pivotal role. As motor coordination improves, fitness components such as strength, endurance, flexibility, and balance advance in tandem, collectively reflecting children’s overall health status (11). In addition, Regular physical activity in preschool years has been positively associated with improvements in executive functions, attention, and academic performance, as evidenced by meta-analytic effect sizes ranging from moderate to large in children (see meta-analysis with g ≈ 0.24–0.90) (18). Systematic reviews focusing on preschool-aged samples further confirm that movement and motor interventions benefit early cognitive and academic skills (19, 20). Despite these advances, relatively few studies have examined population heterogeneity in motor coordination and health-related fitness using combined PCA–cluster approaches. Addressing this gap, the present study investigates the relationship between motor coordination and health-related fitness in preschool children, aiming to provide parents, educators, and policymakers with evidence to guide daily educational practices and physical activity promotion, thereby fostering healthier development in children.

Based on previous evidence, we hypothesize that (1) motor coordination will be positively associated with multiple components of health-related fitness in preschool children; (2) motor coordination will independently predict performance in selected fitness tests even after controlling for body morphology indicators such as BMI; and (3) distinct subgroups of children, characterized by different profiles of motor coordination and fitness, can be identified through cluster analysis. We acknowledge that other potential confounders, such as habitual physical activity and socioeconomic background, were not included in the current analysis but may further influence these relationships.

## 2 Research methods and objects

### 2.1 Research objects

The study participants were recruited from a kindergarten in Weifang, Shandong Province, China. To determine the required sample size, *a priori* calculations were performed using G*Power v3.1 software (power > 0.9, α = 0.05). We assumed a moderate effect size of 0.3, based on meta-analytic finding reporting a moderate to large positive association between motor competence and physical fitness in children(r ≈ 0.43) (21) The required minimum sample size was determined as 111. In total, 374 preschool children were recruited using stratified random sampling, of whom 16 were excluded due to incomplete data. The final analytic sample comprised 358 children (181 boys, 177 girls). Data were collected from October 2023 to January 2024. Descriptive characteristics (height, weight, BMI by gender) are presented in Table 1. In addition to anthropometric data (height, weight, BMI), parental education, socioeconomic background, and children’s habitual physical activity participation were collected through a standardized parent questionnaire. Although not included as primary variables in the present analysis, these demographic factors were documented to provide context and may be explored in future studies. Written informed consent was obtained from all parents or legal guardians prior to participation. The study adhered to the ethical principles of the Declaration of Helsinki and received approval from the Institutional Review Board of the China Institute of Sport Science (Approval No.: CISSLA20230110).

**Table 1:** Sample characteristics.

Variable	Male (*N* = 181)		Female (*N* = 177)	
	Mean	SD	Mean	SD
Height (cm)	111.37	6.88	110.49	7.06
Weight (kg)	19.45	3.93	18.26	3.14
BMI	19.84	4.35	18.32	3.21

### 2.2 Measurement

#### 2.2.1 Motor coordination (MC)

Motor coordination was assessed using the Movement Assessment Battery for Children–Second Edition (MABC-2), Age Band 1, which is specifically designed for children aged 3–6 years. The MABC-2 evaluates three domains of motor performance: manual dexterity, aiming and catching, and balance, across eight standardized test items. Each child was assessed individually in a quiet setting, with testing lasting approximately 30–40 min. Raw scores were converted into age- and gender-adjusted standardized scores, yielding domain scores and a composite standard score reflecting overall motor coordination.

The MABC-2 has been widely used internationally and has been adapted for use in mainland China, with a validated Chinese version and corresponding normative data published in 2016. Psychometric studies have demonstrated strong reliability and validity in preschool populations, including mainland Chinese samples. For example, Hua et al. (9) reported excellent inter-rater and test–retest reliability (ICC ≈ 0.90), acceptable internal consistency, and factorial validity for Age Band 1. These findings support the robustness of the MABC-2 as an appropriate tool for assessing motor coordination in Chinese preschool children.

#### 2.2.2 Physical fitness index (PFI)

Prior to testing commencement, children received demonstrations and explanations of all test requirements. Six physical fitness tests were administered: standing long jump, softball throw, 10-meter shuttle run, 15-meter obstacle run, sit-and-reach, and balance beam walk. Testing strictly adhered to the guidelines stipulated in the National Physical Fitness Measurement (NPFM-preschool children version). This test battery has been validated for use in Chinese preschool populations, demonstrating good reliability and sensitivity across all six items, with intraclass correlation coefficients (ICC_3,1_) in the acceptable to excellent range (0.75–0.88), as well as quantified standard error of measurement (SEM) and minimum detectable change (MDC_95_), thereby supporting its psychometric robustness (22). Scores for the six fitness indicators were standardized as Z-scores according to sex and age group. The specific testing methods were as follows: To account for developmental differences across age and sex, all raw fitness scores were standardized into Z-scores within age- and sex-specific groups. This approach is widely adopted in pediatric physical fitness research to enable comparability across heterogeneous subgroups and minimize confounding by growth-related variability (22). Z-score transformation does not artificially inflate associations but rather normalizes scores to a common scale. Sensitivity analyses using raw scores yielded results consistent with the standardized data, supporting the robustness of this procedure.

##### 2.2.2.1 Standing long jump

Method: The child stood with feet parallel behind the starting line and jumped forward using arm swing; a running start was not permitted. Equipment: Electronic distance-measuring mat. Trials: Two consecutive jumps; the best score was recorded. Unit & Precision: Meters, recorded to two decimal places.

##### 2.2.2.2 Softball throw

Method: The child threw a standard 100 g tennis ball forward using one hand. Trials: Two trials; the best score was recorded. Unit & Precision: Meters, recorded to two decimal places.

##### 2.2.2.3 10-meter shuttle run

Course: A 10-meter straight line was marked on level ground, with start/finish and turn-around lines (approx. 1.5 m wide) drawn at each end. Start gate sensors were placed on either side of the start line. Method: Upon the start command, the child ran to the opposite turn-around line and returned as quickly as possible to the starting line. Equipment: Infrared sensors automatically recorded the time.

##### 2.2.2.4 Trials: one trial

Unit & Precision: Seconds, recorded to one decimal place.15-Meter Obstacle RunCourse: A 15-meter straight line was marked on level ground. A start line (approx. 1.5 m wide) with start gate sensors was positioned at the beginning, and a finish line (approx. 1.5 m wide) with finish gate sensors was positioned at the end. Seven cones were placed along the line: the first cone 3 m from the start, subsequent cones spaced 1.5 m apart (center-to-center), and the final cone 3 m from the finish line. Method: Upon the start command, the child ran as quickly as possible in an S-shaped pattern around the cones to the finish line. Equipment: Infrared sensors automatically recorded the time. Trials: One trial. Unit & Precision: Seconds, recorded to one decimal place.

##### 2.2.2.5 Sit-and-reach

Method: The child sat barefoot on the bench facing the apparatus with legs fully extended, heels together, and soles of the feet flat against the footplate (toes pointing naturally upwards). With hands placed together (palms down) and knees kept straight, the child slowly reached forward with both hands along the measuring scale, pushing the slider as far as possible. Trials: Two consecutive trials; the best score was automatically recorded by the device. Unit & Precision: Centimeters, recorded to one decimal place.

##### 2.2.2.6 Balance beam walk

Method: The child walked the entire length of a balance beam (3 m long, 10 cm wide, 30 cm high). Equipment: Infrared sensors recorded the completion time. Trials: Two trials; the best score was recorded. Unit & Precision: Seconds, recorded to two decimal places.

### 2.3 Procedure

This study included preschool children aged 3–6 years, with data collected in a kindergarten setting. Trained physical fitness assessors conducted the measurements to ensure proficient use of tools and techniques. Testing occurred during spring in a well-lit, spacious indoor area maintained at a comfortable temperature of 22°C. Children wore their regular athletic attire and participated in a 20-min warm-up session involving light exercises and games. To ensure accessibility and minimize fatigue, testing was scheduled simultaneously across multiple mornings between 9:00 AM and 11:00 AM. All assessments followed standardized protocols to maintain consistent indoor conditions and measurement reliability across testing days. Prior to formal testing, an acclimatization session was conducted to familiarize participants with the procedures, reducing potential variability due to unfamiliarity.

### 2.4 Statistical methods

This study first employed descriptive statistical analysis to summarize the basic characteristics of the sample, including means and standard deviations for variables such as height, weight, and BMI. Prior to inferential analyses, data distribution was examined using the Shapiro–Wilk test for normality and Levene’s test for homogeneity of variances. Missing data (<5%) were handled using listwise deletion to avoid bias.

Pearson correlation analysis was then conducted to preliminarily explore the relationships between motor coordination and various physical fitness indicators (e.g., strength, agility, balance), establishing a foundation for deeper investigation. To comprehensively assess the impact of motor coordination on health-related physical fitness, hierarchical linear regression analysis was implemented. In the regression framework, Model 1 included motor coordination as the sole independent variable to evaluate its direct effects on each dimension of physical fitness. Subsequently, Model 2 expanded the analysis by incorporating control variables such as age, gender, and BMI to account for potential confounding factors. Effect sizes and 95% confidence intervals were reported, and Bonferroni corrections were applied to adjust for multiple comparisons. By comparing the two models, the study aimed to isolate the independent influence of motor coordination on fitness outcomes after adjusting for covariates.

To further examine the latent structure of fitness variables, principal component analysis (PCA) was conducted. Sampling adequacy was verified using the Kaiser–Meyer–Olkin (KMO) statistic and Bartlett’s test of sphericity. Component retention was based on eigenvalues >1, scree plot inspection, and theoretical interpretability. Subsequently, cluster analysis (k-means) was applied to classify participants according to their fitness and coordination profiles. The complementarity of PCA and cluster analysis lies in their functions: PCA reduces dimensionality and extracts underlying factors, while clustering classifies individuals based on these factor-derived profiles, thereby ensuring both data simplification and group differentiation. The number of clusters was determined using both the elbow method and silhouette coefficients to ensure robustness of classification. PCA provided dimensional reduction (e.g., agility-dominated vs. strength-dominated dimensions), while clustering identified distinct subgroups, thereby revealing differential patterns of motor competence and fitness.

All statistical procedures were executed using SPSS 27.0 and R 4.3.0, ensuring methodological rigor and reproducibility. This layered analytical approach progressing from descriptive and correlational analyses to advanced multivariate techniques enabled a robust exploration of the complex relationships between motor coordination and health-related fitness in preschool children.

## 3 Results

### 3.1 Correlation analysis

Results of Pearson’s analysis: positive as well as negative correlations were observed between physical fitness variables and motor coordination (Table 2). Positive correlations were observed between the following variables: total motor coordination score was weakly correlated with two-legged consecutive jump (r = 0.184; *p* = 0.001); total motor coordination score was moderately correlated with balance beam (r = 0.211; *p* < 0.01); total motor coordination score was moderately correlated with standing long jump (r = 0.272; *p* < 0.01); total motor coordination score was moderately correlated with the absolute value of the 10-meter toss (r = 0.217; *p* < 0.01); and total motor coordination score was moderately correlated with tennis long jump (r = 0.277; *p* < 0.01). (r = 0.217; *p* < 0.01); total motor coordination score was weakly correlated with tennis ball throw (r = 0.197; *p* < 0.01); and total motor coordination was weakly correlated with seated forward bending (r = 0.125; *p* = 0.024). Total motor coordination score was weakly negatively correlated with BMI (r = −0.232; *p* < 0.01; see Table 3 for details).

**Table 2:** Pearson correlation of body mass index, physical fitness and motor coordination.

Variable	①	②	③	④	⑤	⑥	⑦
① Motor coordination	1						
② Continuous jump on both feet (sec)	0.184** (0.078–0.285,)	1					
③ Balance beam (sec)	0.211** (0.102–0.315)	0.310** (0.204–0.048)	1				
④ Standing long jump (m)	0.272** (0.160–0.378)	0.416** (0.310–0.512)	0.280** (0.159–0.393)	1			
⑤ 10-meter toss (sec)	0.217** (0.116–0.315)	0.404** (0.310–0.490)	0.266** (0.160–0.397)	0.502** (0.409–0.585)	1		
⑥ Tennis ball throw (m)	0.197** (0.095–0.295)	0.178** (0.072–0.280)	0.147** (0.036–0.254)	0.325** (0.215–0.426)	0.284** (0.185–0.378)	1	
⑦ Seated Body Bend (cm)	0.125* (0.016–0.231)	0.065 (−0.047–0.176)	0.052 (−0.065–0.167)	0.157* (0.034–0.275)	0.119* (0.010–0.225)	0.080 (−0.30–0.187)	1
⑧ BMI	−0.232** (−0.342--0.146)	0.014 (−0.268--0.059)	−0.039 (−0.160–0.061)	−0.049 (−0.197–0.037)	0.169** (−0.118–0.091)	−0.272** (−0.342--0.146)	0.243** (−0.048–0.169)

Values represent Pearson’s correlation coefficients (r) with 95% confidence intervals in parentheses. *p* < 0.05; ** *p* < 0.01. False discovery rate (FDR) correction was applied for multiple comparisons; significant correlations after correction are in bold. Post hoc power analyses indicated that all significant correlations had statistical power > 0.90, while non-significant correlations generally showed power < 0.80, suggesting potential Type II error.

**Table 3:** Regression analysis of motor coordination and health related fitness.

Variable	β	*p*	*R* ^2^	Δ*R*^2^	*F*	VIF
Seated forward bending (cm)						
Model 1	0.125	0.024	0.016	0.013	5.121	1.00
Model 2	0.101	0.087	0.008	0.050	0.035	1.00
Continuous jump on both feet (sec)						
Model 1	0.184	0.001	0.034	0.031	11.592	1.00
Model 2	0.198	0.001	0.054	0.040	3.740	1.03
Balance beam (sec)						
Model 1	0.211	0.000	0.045	0.041	14.46	1.00
Model 2	0.246	0.000	0.055	0.039	3.535	1.00
Standing long jump (cm)						
Model 1	0.272	0.000	0.074	0.071	22.093	1.00
Model 2	0.303	0.000	0.137	0.121	8.614	1.01
10-meter dash (sec)						
Model 1	0.217	0.000	0.047	0.045	17.373	1.00
Model 2	0.229	0.000	0.151	0.138	12.276	1.00
Tennis throw (meters)						
Model 1	0.197	0.000	0.039	0.036	14.191	1.00
Model 2	−0.147	0.076	0.116	0.103	9.121	1.01

*F*, *F* statistic; *R*^2^, coefficient of determination (squared); ∆*R*^2^, corrected *R*^2^; *p*, statistical significance; Model 1, motor coordination; Model 2, age, sex, BMI.

### 3.2 Exercise coordination and health-related fitness regression analysis

Stratified regression analysis (Table 2) used similarity-based grouping of variables. The results showed that the *p*-value for Model 1 was less than 0.05 for all test items, indicating that motor coordination had a significant effect on the results of these physical fitness tests. Model 1 had low R^2^ values (0.016–0.074), indicating that motor coordination alone explained a smaller proportion of the variance, and all of the *F*-values were high, indicating that Model 1 was significant overall. In seated forward bends and tennis ball throws, Model (age, gender, and BMI) 2 had higher *p*-values (0.087, 0.076, respectively), indicating that the predictive power of the model was not significant with the addition of age, gender, and BMI in these items. In all other test items, Model 2 had a p-value of 0, indicating significant predictive power of Model 2. The R^2^ values of Model 2 were generally higher than those of Model 1, especially in standing long jump (R^2^ = 0.137) and tossing and running (R^2^ = 0.151), indicating that the explanatory power of the model was significantly improved by adding age, gender, and BMI. The incremental variance explained by the model was greater with the addition of age, gender, and BMI, such as ΔR^2^ = 0.121 for the standing long jump and ΔR^2^ = 0.138 for the tossing and running. The F-values for Model 2 were generally lower than those of Model 1, but remained higher in the tossing and standing long jumps, suggesting that Model 2 was significant overall in these events.

Model 1 had a statistically significant effect (*p* < 0.05) on the seated forward bend, two-legged continuous jump, balance beam, standing long jump, toss and tennis ball throw tests. The second group (height, weight and body mass index) showed statistically significant effect (*p* < 0.05) on two-legged continuous jump, balance beam, standing long jump and tossing and running tests, whereas it did not reach statistical significance (*p* > 0.05) on sitting forward bending and tennis ball toss tests. (see Table 2 for details).

### 3.3 Results of principal component analysis (PCA)

**Figure 1:**
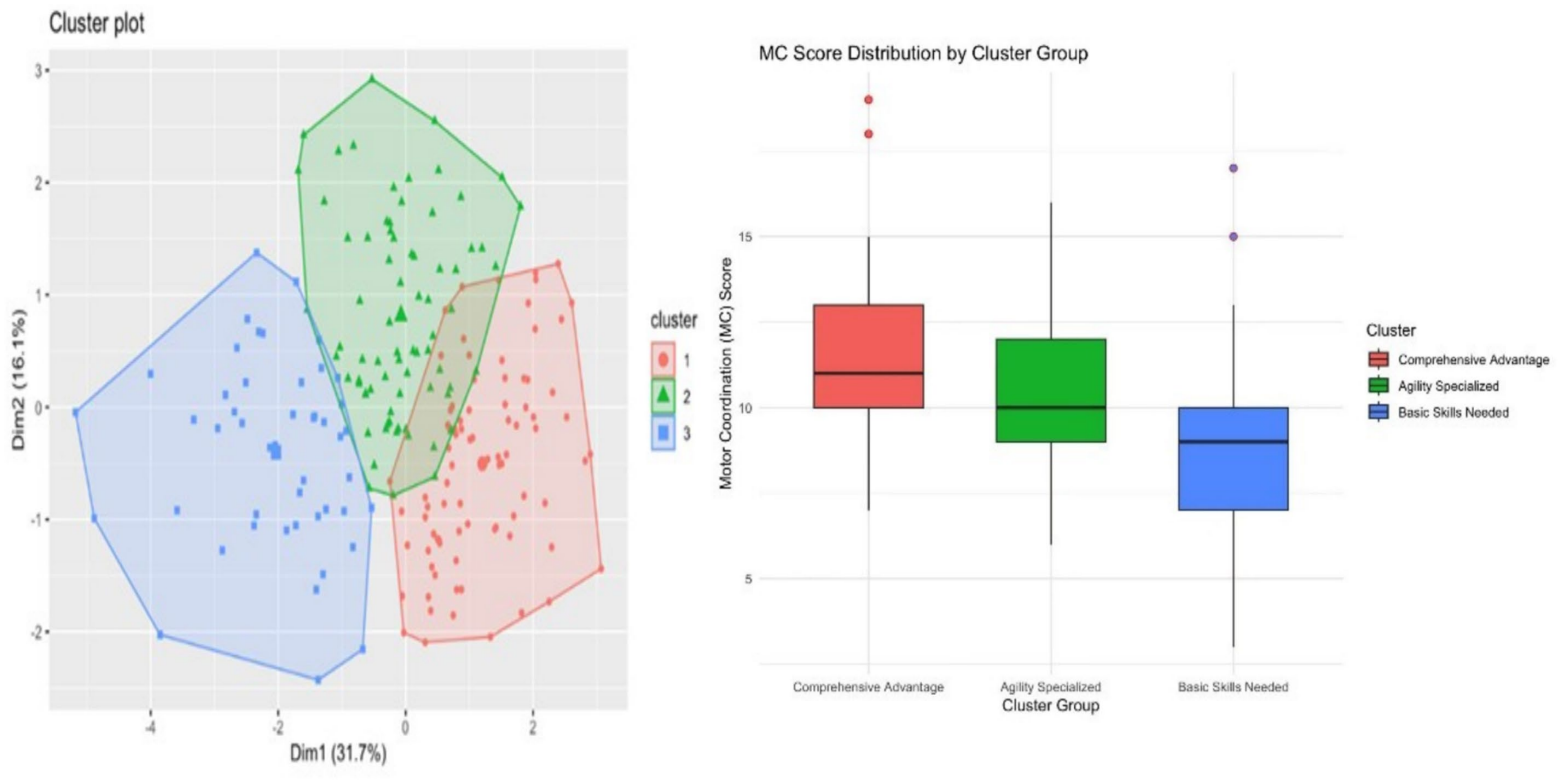
Clustering of preschool children at different levels of development.

Factor loadings indicate the contribution of each fitness variable to the first two PCA components. The silhouette coefficient plot shows that clustering performance was highest when the number of clusters was set to 2, supporting the selection of a two-cluster solution for subsequent analyses. The results of the PCA are shown in Figure 1, indicating that the first two principal components Dim1 and Dim2 together explained 47.8% of the total variance. Dim1 explained 31.7% of the variance, dominated by activities such as running, long_jump, and double_jump, suggesting that these sports played a key role in differentiating between the different groups, while Dim2 explained 16.1% of the variance, dominated by activities such as flexibility and tennis, highlighting the importance of these sports, especially in differentiating between the different groups. tennis, dominated by activities such as flexibility, highlighting the importance of these sports, especially in terms of flexibility, in the delineation of physical fitness groups.

**Figure 2:**
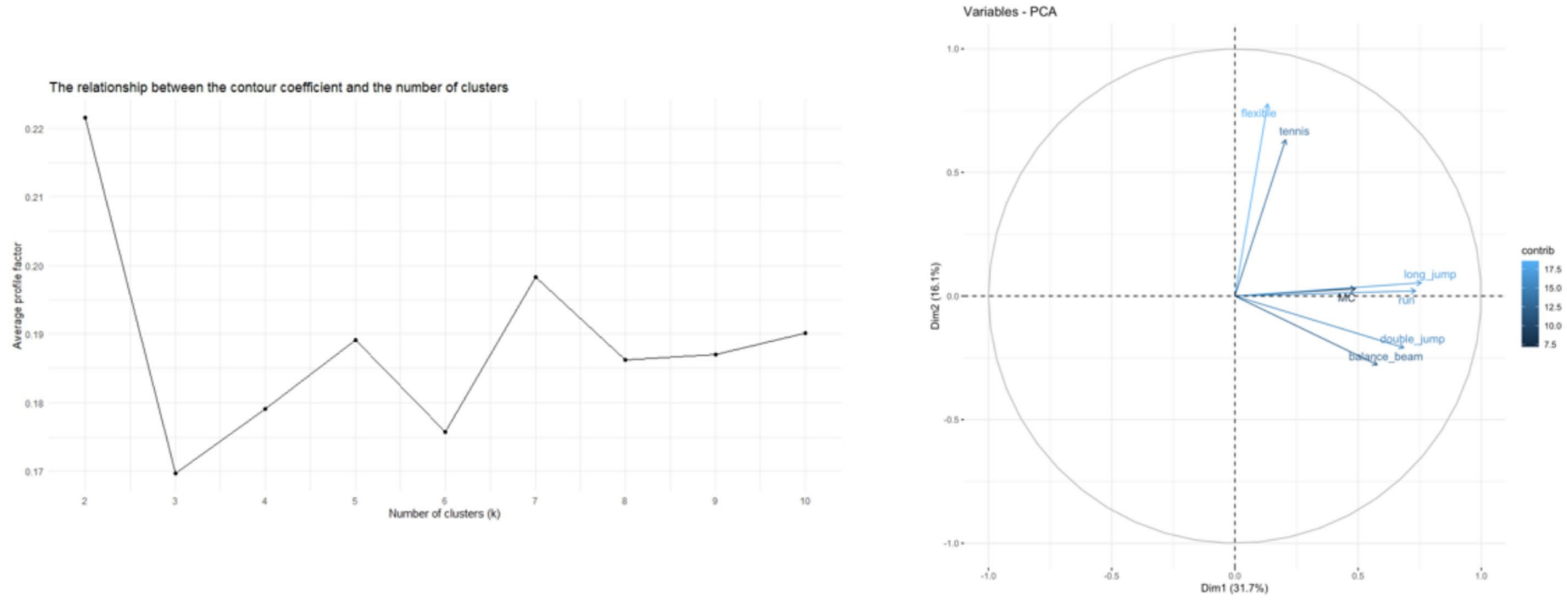
Principal component loadings (left) and silhouette coefficient analysis (right). MC: motor coordination, flexible: seated forward bending, double_jump: two-legged continuous jump, balance_beam: balance beam, long_jump: standing long jump, run: 10-meter dash, tennis: tennis ball throw.

Further analysis of the contribution of each variable to the principal components revealed that running and long jump were highly correlated with Dim1, while flexibility significantly influenced Dim2. According to the factor loadings (see Table 2), the contribution of each variable to the PCA was as follows: the factor loadings for MC (measurement criterion) were Dim1: 0.4899 and Dim2: 0.0301, indicating that its contribution to Dim1 was more prominent; flexibility was more important in the classification of physical fitness groups. The factor loadings for flexibility were Dim1: 0.1330 and Dim2: 0.7773, indicating that its contribution to Dim2 is very important; double_jump had a factor loading of Dim1: 0.6857, reflecting its main contribution to Dim1; balance_beam and running (run) also have a large contribution on Dim1, suggesting that these two variables play an important role in the explanation of physical fitness differences; tennis (tennis) has a high loading on Dim2, emphasizing its role in flexibility and coordination (see Figure 2 and Table 4 for details).

**Table 4:** Principal component analysis (PCA) coefficients.

Variable	Dim.1	Dim.2
MC	0.490	0.030
flexible	0.133	0.777
double_jump	0.686	−0.209
balance_beam	0.578	−0.278
long_jump	0.757	0.0537
run	0.734	0.0209
tennis	0.206	0.632

MC: motor coordination, flexible: seated forward bending, double_jump: two-legged continuous jump, balance_beam: balance beam, long_jump: standing long jump, run: 10-meter dash, tennis: tennis ball throw.

### 3.4 Cluster analysis results

The results of the cluster analysis categorized all samples into three distinct groups (Cluster 1, Cluster 2, and Cluster 3) and showed a clear separation in the PCA plot. Specifically, Cluster 1 (red) is the overall performance excellence group, and this group is more prominent in all types of physical performance indicators. Cluster 2 (green) is the agility expertise group, and this group performs better in sports that require agility, with high flexibility and tennis ability. Cluster 3 (blue), on the other hand, consists mainly of individuals who are weaker in basic skills and who score lower in all measured variables.

The apparent separation of the clustering groups suggests that individuals can be categorized based on their performance in specific physical abilities, such as overall coordination, agility, and foundational skills. A comparison of the box plots of MC scores across the clustering groups revealed that Cluster 1 (Overall Dominance) had the highest MC scores, indicating that individuals in this group possessed strong motor coordination; Cluster 2 (Agility Expertise) showed moderate MC scores, whereas Cluster 3 (Basic Skill Requirements) demonstrated low MC scores.(see Figure 1 for details).

The results of this differential analysis indicate that the three distinct clusters, derived based on motor performance characteristics, exhibited statistically significant differences across all measured motor coordination and physical fitness indicators (*p* < 0.01). This finding validates the effectiveness of the cluster analysis and clearly reveals the characteristic differences in motor capabilities among the identified groups (see Table 5 for details).

**Table 5:** Analysis of differences between clustering groups.

Variable	Cluster 1 (*N* = 84)	Cluster 2 (*N* = 75)	Cluster 3 (*N* = 47)	*p*
MC	11.38 ± 2.45	10.36 ± 2.10	9.15 ± 2.82	<0.01
flexible	0.65 ± 0.77	−0.71 ± 0.85	0.27 ± 0.75	<0.01
double_jump	0.43 ± 0.45	0.38 ± 0.36	−0.91 ± 1.19	<0.01
balance_beam	0.49 ± 0.76	0.10 ± 0.89	−0.63 ± 1.00	<0.01
long_jump	0.88 ± 0.72	−0.09 ± 0.67	−0.72 ± 0.67	<0.01
run	0.65 ± 0.63	0.25 ± 0.60	−0.83 ± 0.88	<0.01
tennis	0.40 ± 1.13	−0.28 ± 0.95	−0.13 ± 1.05	<0.01

## 4 Discussion

This study aimed to explore the relationship between motor coordination and health-related fitness in preschool children and analyze the impact of motor coordination on physical performance. The results revealed significant correlations between motor coordination and multiple fitness indicators, including flexibility, strength, speed/agility, and endurance. Through empirical analysis, combined with principal component analysis (PCA) to extract key components of fitness and motor coordination, cluster analysis further identified distinct groups of children with varying fitness profiles. These findings align with existing research, emphasizing the critical role of motor coordination in children’s physical development (23).

Pearson correlation analysis demonstrated varying degrees of positive correlations between motor coordination and fitness indicators. For instance, moderate positive correlations were observed between motor coordination and tasks such as the double-leg continuous jump, balance beam, standing long jump, and 10-meter shuttle run. This suggests that children with stronger motor coordination typically perform better in tasks requiring balance, explosive power, and agility (24). Similar studies confirm that motor coordination is a key determinant of performance in diverse motor tasks, particularly those demanding precision and rapid responses (25). Additionally, weak positive correlations were found between motor coordination and the tennis ball throw or sit-and-reach test, indicating that coordination also influences flexibility and other physical attributes. The negative correlation with BMI corroborates prior findings, suggesting that higher BMI may impair motor coordination by limiting movement flexibility and precision, thereby compromising overall fitness (26, 27).

Hierarchical regression analysis further validated the significant influence of motor coordination on fitness. Model 1 (motor coordination alone) explained a substantial proportion of variance in fitness outcomes, particularly for standing long jump and shuttle run. While Model 2 (including age, gender, and BMI) improved explanatory power, demographic and body composition variables showed limited significance in certain tests (e.g., sit-and-reach, tennis throw). Overall, motor coordination independently and significantly predicted fitness performance, especially in tasks requiring agility and explosive power (28). These results are consistent with previous studies linking motor coordination, particularly agility, to speed and power in children (29–31).

PCA results highlighted the multidimensional impact of motor coordination on fitness. The first two principal components (Dim1 and Dim2) collectively explained 47.8% of the total variance. Dim1 was dominated by running, long jump, and double-leg jumps, while Dim2 emphasized flexibility and tennis throw. This underscores motor coordination’s role in differentiating fitness profiles, particularly in tasks requiring comprehensive coordination and flexibility. Factor loadings confirmed strong associations between running/jumping and Dim1, whereas flexibility contributed predominantly to Dim2, reflecting the multifunctional role of motor coordination in enhancing diverse fitness attributes.

It is important to note that the first two PCA dimensions accounted for 47.8% of the total variance, leaving a substantial proportion unexplained. This may partly reflect measurement error and unmeasured influences such as daily activity, nutrition, and individual differences. As PCA in this study primarily served as a dimensionality reduction step to support clustering, the moderate variance explained does not undermine the identification of distinct groups. Nevertheless, future studies incorporating broader physiological and environmental variables may better capture unexplained variance.

Cluster analysis classified participants into three groups: “Comprehensive Excellence” (highest motor coordination), “Agility Specialization” (moderate coordination with agility strengths), and “Basic Skill Needs” (low coordination). Comparative analysis revealed significant differences in motor coordination scores across clusters, with Cluster 3 underperforming in running and jumping tasks. These results further validate motor coordination’s positive impact on fitness, particularly in precision- and speed-dependent activities (32–34).

The identification of three distinct clusters highlights heterogeneity in motor coordination and fitness among preschoolers and provides a potential basis for tailored interventions. For example, children in the “Comprehensive Excellence” group may benefit from enrichment opportunities, while those in the “Basic Skill Needs” group may require foundational support. However, these recommendations remain hypothetical. The practical feasibility and effectiveness of cluster-informed interventions should be validated in longitudinal or experimental studies before implementation in educational or clinical contexts.

These findings are consistent with theoretical perspectives in early childhood development, where fundamental motor skills support both physical and cognitive domains (24, 25). Prior research has linked motor coordination to the maturation of brain regions such as the cerebellum, basal ganglia, and prefrontal cortex (26, 27). While our results revealed distinct cluster profiles, suggesting heterogeneity in coordination and fitness, the present cross-sectional design does not allow causal inferences about neurodevelopmental mechanisms. Therefore, PCA-derived dimensions should be interpreted as statistical constructs rather than direct reflections of underlying neural pathways. Longitudinal or neuroimaging studies are needed to clarify the neural bases of these relationships.

These findings are consistent with theoretical perspectives in early childhood development, where fundamental motor skills support both physical and cognitive domains (35, 36). Prior research has linked motor coordination to the maturation of brain regions such as the cerebellum, basal ganglia, and prefrontal cortex (37, 38).

While our results revealed distinct cluster profiles, suggesting heterogeneity in coordination and fitness, the present cross-sectional design does not allow causal inferences about neurodevelopmental mechanisms. Therefore, PCA-derived dimensions should be interpreted primarily as statistical constructs. Nevertheless, drawing on existing literature, these components may tentatively be aligned with neurofunctional processes: Dim1, dominated by running and jumping, could reflect locomotor control supported by cerebellar–basal ganglia pathways, whereas Dim2, emphasizing flexibility and perceptual-motor skills, may correspond to prefrontal-perceptual integration (39).

Importantly, these are hypothetical interpretations, not direct conclusions from our dataset. Longitudinal or neuroimaging studies are needed to validate whether such neural pathways underlie the observed clustering. Still, situating our findings within this developmental framework highlights the potential of motor coordination to serve not only as a marker of physical competence but also as an indicator of broader developmental status (40). The relatively lower coordination and fitness performance in Cluster 3 (“Basic Skill Needs”) underscores the practical importance of early identification and targeted support during sensitive developmental periods (41). In summary, while our study identifies concurrent associations, motor coordination remains a key domain warranting systematic assessment and intervention during the preschool years (42, 43).

In summary, motor coordination not only serves as a core determinant of health-related physical fitness in preschool children but may also act as an observable indicator of their neurodevelopmental status and future potential. Future research should further investigate the mechanisms through which coordination mediates the relationship between physical fitness and cognitive development. It is essential to develop integrated assessment frameworks that combine evaluations of physical fitness, cognitive abilities, and social behaviors, while advancing individualized intervention strategies in practice to promote holistic child development.

## 5 Strengths and limitations

The primary strength of this study lies in its multidimensional assessment approach, integrating statistical methods such as Pearson correlation analysis, hierarchical regression analysis, principal component analysis (PCA), and cluster analysis to comprehensively explore the relationship between motor coordination and health-related fitness in preschool children. The inclusion of a relatively large sample size (N = 358) enhances the statistical reliability of the findings and provides a robust scientific foundation for developing personalized health promotion strategies, demonstrating strong practical value.

However, several limitations should be acknowledged. Most importantly, its cross-sectional design precludes causal inference; the observed associations between motor coordination and fitness cannot establish directionality or underlying mechanisms. Longitudinal and intervention studies are therefore necessary to clarify developmental trajectories. Second, the sample was limited to children from one city in Shandong Province, restricting generalizability. Third, potential confounding factors such as physical activity participation and nutritional status were not controlled. In addition, although acclimatization sessions were conducted, multiple familiarization trials were not systematically implemented, which may have introduced learning-effect biases. Furthermore, data on extracurricular physical activity (e.g., quantity, type, frequency) were not systematically collected; such factors could partly account for the unexplained variance observed in PCA. Future work should address these limitations to strengthen external validity and causal understanding.

## 6 Conclusion

This study revealed significant relationships between motor coordination and health-related fitness in preschool children. Positive associations were found with flexibility, strength, speed/agility, and endurance, while BMI showed a weak negative correlation. Children with stronger motor coordination performed better in tasks requiring balance, explosive power, and rapid responses. Hierarchical regression and principal component analysis (PCA) further confirmed motor coordination as an independent determinant of fitness performance, particularly in agility- and power-demanding tasks. These results highlight motor coordination as a central component of preschool children’s physical development and a potential observable marker of broader developmental status.

Based on these findings, enhancing motor coordination should be prioritized in preschool education and daily activities. At the individual level, playful exercises emphasizing flexibility, balance, and agility can strengthen children’s competence and health outcomes. Parents can support development by providing daily opportunities for both structured and unstructured physical activity. Schools should integrate motor coordination screening into routine health checks to enable early identification and intervention. At the policy level, preschool curricula would benefit from evidence-based guidelines that embed motor and fitness development into early childhood education frameworks.

Future research should adopt longitudinal designs, recruit samples from multiple sites to improve generalizability, and incorporate cognitive outcomes (e.g., executive functions, academic performance) to clarify developmental pathways linking motor coordination, fitness, and broader child development. Expanding the range of environmental and behavioral measures (e.g., extracurricular activity quantity and type) will also help account for unexplained variance observed in PCA.

## Data Availability

The raw data supporting the conclusions of this article will be made available by the authors, without undue reservation.
